# A multimodal individualized long-term intervention to prevent functional decline after stroke (LAST-long): a single blinded randomised controlled trial

**DOI:** 10.1016/j.lanepe.2025.101531

**Published:** 2025-11-13

**Authors:** Torunn Askim, Sara Rise Langlo, Elin Bergh, Øystein Døhl, Hanne Ellekjær, Anne Hokstad, Håkon Ihle-Hansen, Bent Indredavik, Stian Lydersen, Geske Luzum, Yngve Seljeseth, Toril Skandsen, Ingvild Saltvedt, Bente Thommessen

**Affiliations:** a Department of Neuromedicine and Movement Science, Faculty of Medicine and Health Science, NTNU-Norwegian University of Science and Technology, Trondheim, Norway; b Clinic of Internal Medicine, Department of Stroke, St. Olavs Hospital, Trondheim University Hospital, Trondheim, Norway; c Clinic of Rehabilitation, St. Olavs Hospital, Trondheim University Hospital, Trondheim, Norway; d Division of Medicine, Department of Neurology, Akershus University Hospital, Akershus, Norway; e Department of Finance, Trondheim Municipality, Trondheim, Norway; f Department of Medical Research, Bærum Hospital, Vestre Viken Hospital Trust, Bærum, Norway; g Department of Mental Health, Regional Centre for Child and Youth Mental Health and Child Welfare, Faculty of Medicine and Health Science, NTNU-Norwegian University of Science and Technology, Trondheim, Norway; h Department of Medicine, Ålesund Hospital, Helse Møre and Romsdal Hospital Trust, Ålesund, Norway; i Clinic of Internal Medicine, Department of Geriatrics, St. Olavs Hospital, Trondheim University Hospital, Trondheim, Norway

**Keywords:** Cerebrovascular disease, Long-term follow-up, Secondary prevention, Communitybased intervention, Multimodal intervention, Complex intervention, Mixed models, Health Care

## Abstract

**Background:**

Despite the lack of evidence, the Stroke Action Plan for Europe recommends coordinated support after initial rehabilitation. This trial investigated if a coordinator-led multimodal intervention was superior to standard care in preventing dependency and functional decline after stroke.

**Methods:**

In this single blinded randomised controlled multi-centre trial, patients were consecutively screened, included and randomised (1:1) by a web-based randomization system to an intervention or a control group at the out-patient clinics at four Norwegian hospitals three months post-stroke. Blinded assessments were performed at inclusion and at 6-, 12-, and 18-month follow-up. The inclusion criteria were; age ≥18 years, life expectancy ≥12 months (assessed by the physician), modified Rankin Scale (mRS) score <5, vulnerable to functional decline (defined by other measures). The control group received standard care, while the intervention group additionally received monthly follow-up by a community-based stroke coordinator who applied a study specific checklist to identify the patients’ risk profile within multiple domains. Individual goals and action points were defined accordingly. The intervention lasted for 18 months. Mixed models were used to evaluate differences between the groups for the primary endpoint (mRS at 18-month follow-up) and secondary endpoints (activities of daily living, cognitive function, physical function, patient reported outcomes, physical activity, blood pressure, body mass index and blood tests) at 6-, 12-, and 18-month follow-up. The trial was registered at ClinicalTrials.gov identifier: NCT03859063. The trial has been completed.

**Findings:**

The LAST-long trial was conducted from April 11, 2019 to August 28, 2024. In total, 301 participants (48% women); mean age 71·3 (SD 12·3) years, National Institute of Health Stroke Scale admission score 3·38 (SD 4·21), and mRS at inclusion 1·6 (SD 0·9), were randomised to the intervention (n = 152) or control (n = 149) group 108.0 (SD 18·5) days post stroke. The number of serious adverse events (deaths, hospitalisation due to cardiovascular events, cerebrovascular events or fall with or without fracture) was 32 (21·1%) in the intervention group and 33 (22·1%) in the control group. At 18 months, the estimated difference between the groups was 0·03 (95% CI: −0·16 to 0·22), p = 0·79 for mRS.

**Interpretation:**

The 18-month multimodal intervention was not superior to standard care received by the control group in people with mild stroke. An even longer follow-up period might be needed to investigate the effect of the intervention on hard outcomes like death or recurrent vascular events.

**Funding:**

The Research Council of Norway, The Joint Research Committee between St. Olavs Hospital and the Faculty of Medicine and Health Sciences, NTNU (FFU), Foundation Dam.

**Research in context**

**Evidence before this study**

We searched PubMed for articles from inception to April 1, 2025, using the following search terms “stroke (title)”, “multimodal intervention (text word)”, “support programme (text word)”, and “complex intervention (text word)”. There were no language restrictions, however all searches were limited to randomized controlled trials, giving a total of 32 relevant studies. The abstracts were screened carefully, and articles were excluded according to the following criteria: protocol paper, less than 12 months follow-up, unimodal interventions, intervention targeted caregivers and implementation research, leaving 4 relevant articles. From other sources another two trials fulfilling the inclusion criteria were included, giving a total of 6 high quality trials published between 2013 and 2023 available for a thorough review. In all these trials, multiple aspects of post stroke care were considered and coordinated, including secondary prevention, physical activity and other lifestyle factors. Only two trials were published before recruitment to LAST-long commenced by March 2019, while four trials have been published over the past five years. The primary outcomes of these trials were mainly risk factor control and recurrent cerebrovascular disease and only two trials reported functional outcomes as secondary endpoints. The results were inconclusive. While no meta-analyses have been published, a systematic review from 2021 concluded that the included trials were heterogeneous with respect to sample size, duration and design of the intervention program and the choice of outcome measures, giving no clear evidence. In a more recent overview of reviews from 2024 the authors found moderate evidence for multimodal interventions to reduce cardiovascular events and to increase physical activity after stroke, however further high quality RCTs with longer follow-up were requested. Thus, the possible benefit of a long-term coordinated multimodal intervention on functional outcomes remains unclear.

**Added value of this study**

LAST-long is the largest stroke-trial of its kind assessing the impact of an 18-month multimodal approach to long-term follow-up after stroke on functional outcomes. Compared to previous trials, the present trial utilized already existing services in the primary health care system, making the intervention an integrated part of the established clinical practice. The use of the standardized checklist and motivating interviewing to set individual goals and action points in 18 monthly meetings has not been tested earlier. The intervention was safe, while adherence to the intervention was moderate. The neutral results on all functional endpoints indicate that standard care is equal as good as a coordinator-led multimodal intervention for people suffering from mild stroke.

**Implications of all the available evidence**

The Action Plan for Stroke in Europe recommends coordinated support after initial rehabilitation. But given our results, it is too early to conclude that such service should be implemented as part of regular clinical practice for people suffering from mild strokes given that the goal is to improve functional outcomes. Further research is needed to investigate the effect of a coordinator-led multimodal intervention on patient-reported outcomes. Additionally, more qualitative research is needed to explore the stroke-survivors experience with coordinated support and increase the knowledge on how to reach the stroke survivors suffering from moderate to severe disabilities. Furthermore, there is a need for studies with an even longer follow-up period investigating the effect of multimodal interventions on hard outcomes, like cardiovascular events and deaths in a 5- or 10-year perspective.

## Introduction

Functional decline is a major concern in long-term follow-up after stroke.^1^ Despite a significant reduction in age-standardized stroke-related deaths from 1990 to 2021, stroke is still the third most common cause of Disability Adjusted Life Years (DALYs), worldwide.^2^

Great achievements have been reached within acute stroke treatment over the past decades with strong evidence for treatment in an organized stroke unit and reperfusion therapy for patients with acute ischemic stroke. There is also strong evidence for multidisciplinary person-centred rehabilitation and early supported discharge.^3^ In addition, immediate and sustained implementation of effective and appropriate secondary prevention strategies has the potential to reduce the risk of recurrent stroke significantly.^4^ Secondary prevention should include regular use of recommended medication and lifestyle changes to reduce the risk of recurrent cardiovascular events and preserve function.^4^^,^^5^ Engagement in physical activity is critically important, as structured exercise has dual benefits by both reducing the risk of recurrent stroke and enhancing overall function^6^ beyond the functional decline expected in older adults.^7^ Nevertheless, there is substantial variation in adherence to the guidelines, especially in the long term.^8^ There is also a need for research on the transition from rehabilitation to life after stroke. Therefore, the Stroke Action Plan for Europe emphasizes long-term follow-up as one of the research priorities toward 2030, and identification of an optimal model of care and long-term support after stroke.^9^

Despite the lack of a universally accepted definition, multimodal interventions are commonly understood as interventions that aim to ensure that multiple aspects of secondary prevention and life after stroke are being considered and coordinated. So far, the results from such studies are inconclusive.^10^, ^11^, ^12^, ^13^, ^14^ A systematic review from 2021 identified 41 randomised controlled trials aiming to improve long-term management following stroke.^15^ The included trials were heterogeneous with respect to sample size, duration and design of the intervention program, and choice of outcome measures, giving no clear evidence, and the authors ask for further high-quality RCTs with both clinical and patient-reported outcome measures.^15^ In a more recent overview of reviews from 2024, the authors found moderate evidence for multimodal interventions to reduce cardiovascular events and to increase physical activity after stroke. Still, they concluded that further high-quality RCTs with longer follow-up were required.^16^

The most recent cluster-randomised controlled trial investigated the effectiveness of a structured ambulatory post-stroke care program with regular follow-up.^17^ The intervention was tailored to patients’ individual needs and aimed to personalize the follow-up. No significant effect on one year mortality was observed, despite improved cardiovascular risk control, while secondary endpoints like independence, activities of daily living (ADL) and cognition have not been published yet.^17^ Nevertheless, the authors suggested that the 1-year follow-up period was too short to assess the long term effects and a longer follow-up period may be needed to evaluate the effect of a multimodal intervention on such outcomes. Therefore, the primary objective of the LAST-long trial was to investigate whether an 18-month multimodal personalized intervention delivered by a dedicated stroke-coordinator was superior to standard care with respect to dependency. Secondary objectives were to investigate whether the intervention was superior to standard care with respect to activities of daily living, cognitive function, physical function, physical activity, patient reported outcome measures (PROMS) and secondary prevention measures, like blood samples, blood pressure and body mass index.

Our primary hypothesis was.

- The LAST-long intervention is superior to standard post-stroke care in preventing dependency, as measured by the modified Rankin Scale (mRS) at 18-month follow-up.

Our secondary hypothesis was.

- The LAST-long intervention is superior to standard post-stroke care in preventing decline in activities in daily living, cognitive function, physical function, physical activity, patient reported outcome measures and secondary prevention outcome measures at 18-month follow-up.

## Methods

### Study design

A pragmatic single-blinded, parallel-group randomized controlled multicentre trial was conducted at four centres in Norway; including two university hospitals and two general hospitals. The trial was conducted in close collaboration with the primary healthcare services of six municipalities with a population of 45,000 to 200,000 inhabitants. Follow-up assessments were carried out at each hospital at 6, 12, and 18 months after inclusion.

Ethical approval was granted by the Regional Committee of Medical and Health Research Ethics Central Norway, REC no. 2018/1809 on January 22, 2019 (See details in Supplementary Material). In line with the Declaration of Helsinki, written consent was obtained from all participants at the time of inclusion before enrolment. The protocol was published and the trial was registered at Clinicaltrial.gov, http://www.clinicaltrials.gov/study/NCT03859063. The trial has been completed.

This trial was not monitored by a Data and Safety Monitoring Board (DSMB). Due to Norwegian health research regulations, it is not mandatory to set down a DSMB for trials that do not include testing of new drugs or medical devices.

### Participants

Individuals living in one of the participating municipalities who were admitted to hospital with the diagnosis of first ever or recurrent stroke (ischemic stroke or haemorrhage) were screened for inclusion and recruited into the trial at the out-patient clinics 2–4 months post-stroke, according to the following criteria: Age ≥18 years, modified Rankin Scale (mRS) < 5, able to understand the Norwegian language and able and willing to sign informed consent. In addition, participants were required to have a persistent impairment following stroke, as indicated by at least one of the following criteria: <10 points on Short Physical Performance Battery (SPPB) or <26 points on Montreal Cognitive Assessment (MoCA) or >27 points on the 7-item version of the Fatigue Severity Scale (FSS-7) or >7 points on the anxiety items on Hospital Anxiety and Depression Scale (HADS) or >7 points on the depression items of HADS or not being able to draw 10 horizontal lines within 20 s (item 3 on Advanced Hand Activities, Motor Assessment Scale).

Individuals were excluded if their life expectancy was <12 months (assessed by the physician), if they had other serious diseases that made it difficult to comply with the intervention (i.e., serious neurological diseases, dementia, or drug abuse) or if they were already included in another intervention study.

### Randomisation and masking

Individuals who fulfilled all inclusion criteria performed all baseline tests prior to randomization. Randomization was performed by a web-based randomization system developed and administered by the Clinical Research Unit, Faculty of Medicine and Health Science, Norwegian University of Science and Technology. Participants were stratified according to age (>80 years), dependency level (mRS <3), and hospital site. They were randomly assigned (1:1), in blocks of varying and unknown size, to the intervention group or the control group. Assessors blinded to group allocation performed assessments at 6-, 12-, and 18-month follow-ups.

### Procedures

LAST-long was performed within the context of the Norwegian public health service. This covers all levels of hospital services, rehabilitation, and primary health care including general practitioners (GPs), physiotherapists, home care services, and nursing homes. The public health care in Norway is organised through four regional health authorities. These provide most hospital services and cover rehabilitation services to inhabitants within their respective regions. GPs and physiotherapists outside hospitals are partly reimbursed by governmental means. Otherwise, each municipality funds, organizes, and manages all primary health services like community-based rehabilitation, home-based care, and nursing homes.

All participants in LAST-long received evidence-based stroke unit treatment at the hospital in the acute phase and further rehabilitation according to individual needs after discharge. The rehabilitation usually consists of inpatient rehabilitation, rehabilitation in the patient's home, or at an outpatient clinic. It is usually limited to the first 3 months for patients with mild to moderate strokes but can last for up to 6 months for patients with the most severe strokes and for selected patients even longer.

Patients were screened for inclusion at the compulsory outpatient consultation 3 months after stroke. Well-trained study personnel performed the screening procedure and baseline testing before randomization, in that specific order on the day of inclusion. At the 6-, 12- and 18-month follow-up assessments, study personnel blinded to group allocation performed the comprehensive test battery.

Participants randomised to the control arm received standard care only, while participants randomised to the intervention arm received long term follow-up by a new established community-based stroke-coordinator within their municipality in addition to standard care. The coordinators had a professional background as either physiotherapist, occupational therapist or nurse. The main role of the coordinator was twofold; 1) to set up an individualized treatment plan and 2) to motivate and assist in getting access to the existing services. Further details of the intervention have been described in the protocol paper^18^ and in the Supplementary Material (See Supplementary TIDieR Checklist). The intervention is also summarized below:

A study-specific checklist (the LAST-long checklist) was developed based on the World Stroke Organisation (WSO) Post-Stroke Checklist^19^ and Comprehensive Geriatric Assessment (CGA).^20^ The original CGA includes four domains of assessment that are; medical assessment, assessment of function, assessment of cognition and mood and social assessment, and is shown to be effective for improving survival and function in older persons. On the other hand, the WSO Post Stroke Checklist, addressing 11 areas, is also aiming to improve long-term stroke care. In the LAST-long checklist, the domains from the WSO Post Stroke Checklist and the CGA were combined to cover all relevant areas for long-term follow-up after stroke.

In the present study, the LAST-long checklist was used as a guide for a structured interview to assess the patients’ risk profile within the following domains: 1) health and lifestyle, 2) physical function, 3) cognition and mood, and 4) social function.^18^

For patients with risk of deterioration within one or more domains, the stroke-coordinator and the participant agreed on an appropriate treatment plan, based on individual goals, aiming to maintain or improve function. The plan consisted of 2–3 achievable goals and a corresponding treatment plan making use of already available community-based or hospital-based services as recommended by the stroke-coordinator. Within the domain of health and lifestyle factors the goals were based on recommended guidelines^5^ Within the domains of physical function, cognition and mood and social function where no guidelines existed, best evidence and good clinical practice were used to tailor goalsetting and the action plan. An example of a treatment plan has been illustrated in Supplementary Material (See Supplementary Figure S1).

Regular workshops were arranged for the coordinators to ensure adherence to the intervention protocol. In addition, the stroke-coordinators were certified in motivational interviewing as a technique to facilitate lifestyle changes and improve adherence to the treatment plan. At each follow-up meeting, the previous goal and treatment plan were evaluated, and a revised plan was set for the next month. The follow-up consisted of 18 monthly meetings between the participant and the stroke-coordinator, allowing for up to 50% of the meetings being phone-meetings. In line with previous multimodal intervention trials, an overall attendance rate of 75% was defined as good adherence.^18^

The first five included participants were defined as pilot participants. However, no significant adjustments were made accordingly, and they were therefore included in all analysis.

### Outcomes

This trial was aiming to prevent decline in global function and maintain independence in daily living. Hence, mRS at 18-month follow-up was defined as the primary outcome.^21^

Due to the restriction during the pandemic and other reasons for not being able to show up in-person for testing, we allowed for telephone interviews and in a few cases information was collected from a proxy for the primary outcome and self-reported outcomes throughout the trial.

In addition, activities of daily living (ADL) was measured by Barthel Index while instrumental ADL ability was measured using the Nottingham Extended ADL scale (NEADL).

Global cognitive function was measured by the Montreal Cognitive Assessment (MoCA) and the Global Deterioration Scale (GDS). Complex attention and executive function were measured using Trail Making Test A and B (TMT A and B).

The Short Physical Performance Battery (SPPB), consisting of three subtasks: gait speed, balance, and chair rise, was used as a measure of physical performance, while the Six-minute-walk-test (6MWT) was used as a measure of physical capacity, using the standard test procedure in a 10-m walkway. Grip strength was measured by a Jamar handhold dynamometer. The patient reported outcome measures (PROMS) included the first-level EuroQol five-dimensional descriptive system (EQ-5D-5L), Stroke Impact Scale (SIS), the 7-item version of Fatigue severity scale (FSS-7) and Hospital Anxiety and Depression Scale (HADS). In addition, the average number of steps per 24-h were collected from the ActivPAL activity monitor. Furthermore, systolic blood pressure, body mass index and blood samples (total cholesterol, low density lipid, HbA1C and CRP) were collected as measures of secondary prevention. The details of all outcome measures have been reported in the protocol paper.^18^ In the protocol paper, caregivers’ burden and the use of health services were also listed as secondary outcomes. Since these outcome variables were intended to be used in a future cost-effectiveness analysis, they will be published in a separate paper at a later stage.

Study personnel, specially trained in administering both the primary and all secondary outcome measures, conducted assessments at each follow-up timepoint.

Even though, the LAST-long intervention was defined as a low-risk lifestyle intervention, information about Serious Adverse Events (SAEs) were collected retrospectively from the patients’ hospital records. SAEs were defined as hospitalisation due to falls (with or without fracture), cardiovascular events (myocardial infarction, atrial fibrillation or heart failure) or cerebrovascular events (cerebral infarction, cerebral haemorrhage or transitory ischemic attack) occurring during follow-up.

Adherence to follow-up by the stroke-coordinator was obtained by registering the number of attended meetings and the content of these. Furthermore, goal achievement and adherence to the action plan was evaluated in every meeting unless in the first meeting. For this purpose, we asked the participants to rate on a numeric rating scale (1 = fully disagree to 5 = fully agree) to which extent they agreed to the following statements; 1) I achieved my goals, 2) I adhered to the action plan.

### Statistical analysis

The sample size calculation was based on data from the previous LAST study,^22^ with an estimated mean (SD) deterioration of 0·4 (1·47) points on mRS in the control group and maintenance of function 0·0 (1·44) points in the intervention group. We estimated the correlation between mRS at baseline and 18 months to be R = 0·445, giving R squared = 0·20. With 150 participants in each of the two groups, this gives a power of 76% at a significance level of 0·05. Further details about the sample size estimation are outlined in the Supplementary Material (see details in the Supplementary Material).

As stated in the statistical analysis plan (SAP) in the protocol paper,^18^ mixed models were used to evaluate differences between the intervention and control group for the primary and secondary endpoints across the four time points, with time as categorical covariate and its interaction with treatment group as fixed effects, and patient as random effect. The coefficient of this interaction is reported as the treatment effect. The baseline value of the dependent variable was handled as recommended by Coffman et al., 2016.^23^ The mixed model includes participants with outcome data on at least one time point, hence the primary analysis was by intention to treat. The estimates are unbiased if data are missing at random (MAR), while a complete case analysis would be unbiased only under the more restrictive missing completely at random (MCAR) assumption. The model was adjusted for the variables used in the stratified randomization (age, dependency level, and hospital site), as well as sex and the admission National Institute of Health Stroke Scale (NIHSS) score as a measure of stroke severity.

Mixed models were also used in the post-hoc sensitivity analysis, assessing the differences between the groups including only participants with good adherence to the intervention, i.e., attending at least 75% of the meetings (14 out of 18 meetings) in the intervention group.

Finally, mixed models were used for the post-hoc subgroup analyses for all covariates: sex (female versus male), stroke severity (NIHSS score <8 versus ≥8), pre-stroke function (mRS 0–2 versus 3–4) and hospital site (Hospital 1, 2, 3 and 4) with mRS at 6, 12 and 18 months as the outcome variables.

### Role of the funding source

The funders of the study had no role in study design, data collection, data analysis, data interpretation, or writing of the report.

## Results

A total of 2528 stroke patients residing in one of the six participating municipalities were admitted to one of the four collaborating hospitals between January 1, 2019, and November 30, 2022. The commencement of recruitment was consecutive, with the final hospital starting to recruit in November 2021.

**Fig. 1:**
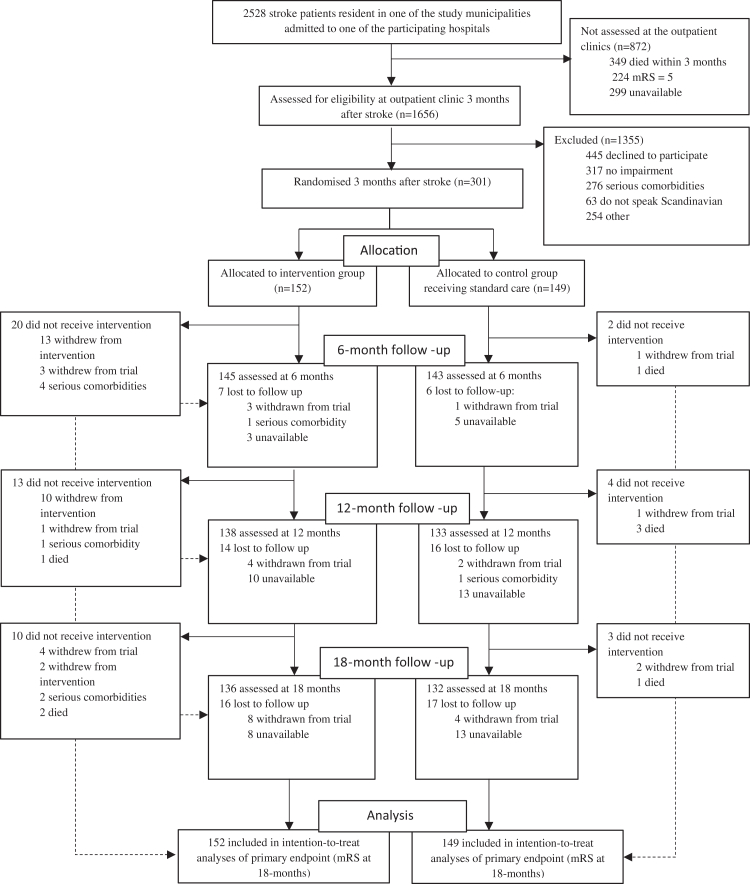
Flow of participants through the trial.

At the outpatient clinics, three months after the stroke, 1656 patients were screened for inclusion. Details about exclusion and the flow of participants through the trial are shown in Fig. 1. The follow-up period was completed by the end of August 2024.

Demographic and baseline characteristics were similar in both groups (Table 1).

**Table 1:** Baseline characteristics.

	Intervention (n = 152)	Control (n = 149)	Total sample (n = 301)
Age (years), mean (SD)	70·7 (13·0)	71·9 (11·6)	71·3 (12·3)
>80	40 (26·3)	36 (24·2)	76 (25·2)
Sex^a^			
Female	67 (44·1)	77 (51·7)	144 (47·8)
Male	85 (55·9)	72 (48·3)	157 (52·2)
Ethnicity^b^			
Discharged to			
Home with or without care	85 (55·9)	80 (53·7)	165 (54·8)
Home with rehabilitation	11 (7·2)	17 (11·4)	28 (9·3)
Inpatient rehabilitation	49 (32·2)	46 (30·9)	95 (31·6)
Short stay in nursing home	4 (2·6)	6 (4·0)	10 (3·3)
Another hospital ward	3 (2·0)	0 (0·0)	3 (1·0)
NIHSS admission, mean (SD)	3·5 (4·7)	3·3 (3·7)	3·4 (4·2)
<8	132 (86·8)	131 (87·9)	263 (87·4)
8–16	13 (8·6)	16 (10·7)	29 (9·6)
>16	7 (4·6)	2 (1·3)	9 (3·0)
Affected side of the body			
Right	59 (38·8)	59 (39·6)	118 (39·2)
Left	62 (40·8)	59 (39·6)	121 (40·2)
Both	8 (5·3)	14 (9·4)	22 (7·3)
None	2 (1·3)	1 (0·7)	3 (1·0)
Stroke type			
Infarction	142 (93·4)	135 (90·6)	277 (92·0)
Haemorrhage	8 (5·3)	13 (8·7)	21 (7·0)
Unknown (neg MR/CT)	2 (1·3)	1 (0·7)	3 (1·0)
TOAST classification			
Large-artery atherosclerosis	29 (19·1)	23 (15·4)	52 (17·3)
Cardio embolism	27 (17·8)	28 (18·8)	55 (18·3)
Small-vessel occlusion	28 (18·4)	34 (22·8)	62 (20·6)
Stroke of other determined ethology	13 (8·6)	7 (4·7)	20 (6·6)
Stroke of undetermined ethology	47 (30·9)	44 (29·5)	91 (30·2)
Haemorrhage	8 (5·3)	13 (8·7)	21 (7·0)
Pre-stroke mRS, mean (SD)	0·62 (0·80)	0·84 (0·94)	0·73 (0·88)
Pre-stroke mRS, median (IQR)			0 (0–1)
mRS = 0	84 (55·3)	72 (48·3)	156 (51·8)
mRS = 1	46 (30·3)	36 (24·2)	82 (27·3)
mRS = 2	18 (11·8)	34 (22·8)	52 (17·3)
mRS = 3	4 (2·6)	7 (4·7)	11 (3·7)
Pre-stroke comorbidity			
Cerebral infarction	27 (17·8)	28 (18·8)	55 (18·3)
Cerebral haemorrhage	7 (4·6)	5 (3·4)	12 (4·0)
TIA	13 (8·6)	15 (10·1)	28 (9·3)
Myocardial infarction	21 (13·8)	21 (14·1)	42 (14·0)
Heart failure	6 (3·9)	3 (2·0)	9 (3·0)
Atrial fibrillation	22 (14·5)	21 (14·1)	43 (14·3)
Hypertension	77 (50·7)	82 (55·0)	159 (52·8)
Hypercholesterolemia	57 (37·5)	47 (31·5)	104 (34·6)
Diabetes	30 (19·7)	21 (14·1)	51 (16·9)
Severe chronic pulmonary disease	10 (6·6)	10 (6·7)	20 (6·6)
Kidney disease	5 (3·3)	11 (7·4)	16 (5·3)
Systemic inflammatory disease	12 (7·9)	2 (1·3)	14 (4·7)
Cancer	13 (8·6)	13 (8·7)	26 (8·6)
Education, mean (SD) years	11·3 (2·9)	11·6 (2·8)	11·4 (2·9)
≤12 years, n (%)	87 (57·2)	84 (56·4)	171 (56·8)
At inclusion (3 months post stroke)			
Time from stroke (days), mean (SD)	109·3 (17·1)	106·8 (19·8)	108·1 (18·5)
Living situation			
Alone	50 (32·9)	54 (36·3)	104 (34·6)
mRS, mean (SD)	1·62 (0·91)	1·66 (0·86)	1·64 (0·89)
mRS, median (IQR)	2 (1)	2 (1)	2 (1–2)
mRS = 0	11 (7·2)	10 (6·7)	21 (7·0)
mRS = 1	64 (42·1)	56 (37·6)	120 (39·9)
mRS = 2	55 (36·2)	59 (39·6)	114 (37·9)
mRS = 3	16 (10·5)	22 (14·8)	38 (12·6)
mRS = 4	6 (3·9)	2 (1·3)	8 (2·7)
Number recruited at each hospital site			
Hospital 1	45 (49·45)	46 (50·55)	91 (30·2)
Hospital 2	68 (50·00)	68 (50·00)	136 (45·2)
Hospital 3	34 (50·75)	33 (49·25)	67 (22·3)
Hospital 4	5 (71·43)	2 (28·57)	7 (2·3)

Values are n (%) unless stated otherwise.

NIHSS = The National Institutes of Health Stroke Scale, mRS = Modified Rankin Scale, MMSE = Mini-Mental State Examination, SD = Standard deviation, IQR = Interquartile range, MR = Magnetic resonance, CT = computer tomography.

a Self-reported.

b Ethnicity data were not collected because it was not expected to be a significant risk factor in this study.

**Fig. 2:**
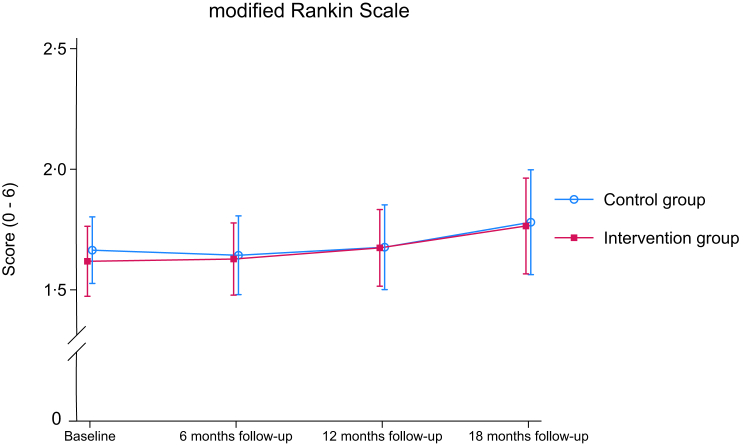
Mean change in modified Rankin Scale (mRS) by group from baseline to 18-month follow-up.

Mean mRS changed from 1·62 (SD 0·91) to 1·76 (SD 1·18) from baseline to 18-month follow-up in the intervention group and from 1·66 (SD 0·86) to 1·78 (SD 1·27) in the control group (Table 2 and Fig. 2). The estimated difference between intervention and control group was 0·03 (95% CI: −0·16 to 0·22) points (p = 0·79) at 18 months.

**Table 2:** Primary and secondary outcomes.

	Intervention (n = 152)			Control (n = 149)			Difference (Group x Time)^a^		
	n	Mean	SD	n	Mean	SD	Estimate	95% CI	p-value
Modified Rankin Scale (mRS)									
Baseline	152	1·62	0·91	149	1·66	0·86			
6 months	145	1·63	0·92	143	1·64	1·00	0·01	−0·17 to 0·20	0·88
12 months	138	1·67	0·95	133	1·68	1·03	0·01	−0·18 to 0·20	0·93
18 months	136	1·76	1·18	132	1·78	1·27	0·03	−0·16 to 0·22	0·79
Barthel Index									
Baseline	151	95·70	9·04	147	97·24	5·89			
6 months	136	96·51	7·23	137	96·46	6·45	0·28	−1·10 to 1·67	0·69
12 months	130	96·54	7·28	122	96·11	8·44	0·95	−0·49 to 2·38	0·20
18 months	122	95·70	10·13	118	96·73	7·26	−0·92	−2·39 to 0·55	0·22
Nottingham Extended ADL Scale									
Baseline	152	52·82	12·39	148	54·16	11·50			
6 months	132	56·04	11·06	134	56·44	10·60	−0·16	−1·90 to 1·57	0·86
12 months	125	56·65	12·07	118	57·16	11·30	0·46	−1·35 to 2·26	0·62
18 months	119	56·97	12·73	113	55·50	11·44	1·09	−0·75 to 2·93	0·25
Global Deterioration Scale									
Baseline	152	2·10	0·95	149	2·17	0·95			
6 months	138	2·14	1·08	132	2·16	1·01	0·09	−0·10 to 0·28	0·36
12 months	126	1·93	1·03	120	2·06	1·0	−0·06	−0·25 to 0·14	0·57
18 months	118	1·98	1·13	110	2·02	1·04	0·12	−0·08 to 0·33	0·23
Montreal Cognitive Assessment (MoCA)									
Baseline	151	24·15	4·00	149	24·36	3·67			
6 months	124	24·65	3·74	119	24·92	3·83	−0·50	−1·13 to 0·14	0·12
12 months	111	25·66	3·81	109	25·35	3·93	0·19	−0·46 to 0·85	0·57
18 months	104	25·97	3·37	97	25·16	4·33	0·26	−0·42 to 0·94	0·45
Trail Making Test Part A (TMT-A)									
Baseline	148	55·11	34·69	146	59·23	38·68			
6 months	123	52·15	28·55	115	54·87	35·47	0·67	−5·44 to 6·77	0·83
12 months	110	52·0	32·47	107	55·60	36·95	0·33	−6·02 to 6·69	0·92
18 months	103	45·76	25·42	95	58·07	45·43	−3·62	−10·23 to 2·99	0·28
Trail Making Test Part B (TMT-B)									
Baseline	137	145·22	85·45	127	139·85	82·86			
6 months	114	148·0	85·12	107	154·41	94·71	−4·09	−19·30 to 11·13	0·60
12 months	102	141·85	84·87	95	144·54	84·11	−2·67	−18·66 to 13·33	0·74
18 months	95	125·76	78·19	87	149·86	90·59	−14·08	−30·61 to 2·45	0·095
Short Physical Performance Battery (SPPB)									
Baseline	151	9·81	2·72	147	9·88	2·59			
6 months	121	9·49	2·72	120	9·33	2·60	0·21	−0·24 to 0·65	0·36
12 months	112	9·58	2·28	109	9·29	2·79	−0·11	−0·57 to 0·35	0·63
18 months	104	9·60	2·62	97	9·53	2·52	−0·34	−0·82 to 0·14	0·16
Six Minute Walk Test (6MWT)									
Baseline	125	370·77	109·78	123	361·39	123·36			
6 months	106	361·35	135·29	110	365·0	134·93	−13·71	−30·56 to 3·12	0·11
12 months	103	386·62	131·79	102	361·79	139·35	6·10	−11·11 to 23·32	0·49
18 months	93	396·65	124·96	90	366·41	133·63	6·35	−11·53 to 24·23	0·49
Grip strength right									
Baseline	149	29·8	14·0	145	28·85	11·86			
6 months	116	29·36	14·20	117	28·85	12·09	−0·10	−1·43 to 1·24	0·89
12 months	112	29·95	14·11	110	28·99	12·78	0·12	−1·24 to 1·48	0·86
18 months	103	29·98	14·09	96	29·25	13·07	0·13	−1·29 to 1·55	0·86
Grip strength left									
Baseline	150	27·75	12·64	144	25·88	12·37			
6 months	119	27·45	13·15	117	25·48	12·29	0·63	−0·64 to 1·87	0·33
12 months	113	29·24	12·72	110	25·78	12·88	0·86	−0·43 to 2·15	0·19
18 months	101	29·28	12·37	96	25·67	13·36	0·96	−0·40 to 2·32	0·17
Fatigue Severity Scale (FSS)									
Baseline	152	3·548	1·99	148	3·427	2·09			
6 months	135	3·713	1·77	132	3·789	1·87	−0·176	−0·531 to 0·180	0·333
12 months	127	3·522	1·75	119	3·411	1·86	−0·037	−0·406 to 0·331	0·843
18 months	116	3·730	1·83	108	3·389	1·91	0·166	−0·218 to 0·550	0·396
Hospital Anxiety and Depression Scale, total (HADS)									
Baseline	152	6·658	6·16	148	5·905	5·77			
6 months	136	8·419	5·99	132	7·992	6·20	0·0103	−1·035 to 1·055	0·984
12 months	128	8·117	6·46	119	8·067	5·79	−0·426	−1·507 to 0·655	0·440
18 months	115	7·991	6·19	107	7·150	6·01	0·146	−0·984 to 1·276	0·800
Hospital Anxiety and Depression Scale, Anxiety (HADS-A)									
Baseline	152	3·533	3·64	148	3·297	3·73			
6 months	136	4·257	3·61	132	3·992	3·79	0·164	−0·488 to 0·816	0·622
12 months	128	4·086	3·63	119	4·151	3·70	−0·094	−0·770 to 0·581	0·784
18 months	115	4·035	3·49	108	3·648	3·73	0·062	−0·643 to 0·766	0·864
Hospital Anxiety and Depression Scale, Depression (HADS-D)									
Baseline	152	3·125	3·24	148	2·608	2·63			
6 months	136	4·161	3·55	132	4·0	3·32	−0·132	−0·725 to 0·460	0·662
12 months	128	4·031	3·69	119	3·916	2·94	−0·310	−0·924 to 0·304	0·322
18 months	115	3·957	3·52	107	3·467	3·15	0 ·128	−0·513 to 0·769	0·696
EQ-5D-5L index									
Baseline	152	0·831	0·17	149	0·838	0·16			
6 months	133	0·806	0·18	132	0·824	0·17	−0·016	−0·469 to 0·016	0·328
12 months	127	0·863	0·17	122	0·827	0·17	0·011	−0·021 to 0·043	0·514
18 months	119	0·847	0·01	113	0·851	0·02	0·002	−0·031 to 0·035	0·901
EQ-5D-5L VAS									
Baseline	149	67·832	17·11	148	65·783	19·59			
6 months	133	65·586	18·17	131	67·679	18·33	−3·434	−7·045 to 0·175	0·062
12 months	123	70·455	17·0	118	68·483	18·03	0·153	−3·604 to 3·910	0·936
18 months	113	70·50	17·26	110	70·41	18·89	−1·549	−5·434 to 2·335	0·434
Stroke Impact Scale (SIS), over all recovery									
Baseline	148	70·864	20·38	147	70·612	21·18			
6 months	134	72·764	22·03	131	70·473	24·28	0·614	−3·703 to 4·930	0·780
12 months	126	72·968	23·80	119	72·050	24·36	0·213	−4·253 to 4·679	0·926
18 months	113	73·708	21·60	109	72·606	22·82	−1·508	−6·163 to 3·147	0·525
Stroke Impact Scale (SIS), Strength									
Baseline	148	79·899	21·32	144	80·816	23·57			
6 months	132	78·125	24·65	128	79·248	23·45	0·545	−3·351 to 4·440	0·784
12 months	125	80·950	22·70	119	81·670	22·32	0·710	−3·301 to 4·721	0·729
18 months	114	78·783	22·10	107	81·133	22·45	−1·722	−5·900 to 2·455	0·419
Stroke Impact Scale (SIS), Hand function									
Baseline	151	83·046	24·73	145	85·034	22·62			
6 months	131	83·817	24·49	126	86·627	22·21	−0·794	−4·011 to 2·423	0·629
12 months	123	84·797	24·90	118	86·102	24·38	0·923	−2·382 to 4·229	0·584
18 months	115	87·696	19·55	108	86·435	24·50	0·780	−2·628 to 4·187	0·654
Stroke Impact Scale (SIS), Activities of Daily Living/Instrumental Activities of Daily Living									
Baseline	151	87·431	15·88	144	88·527	13·50			
6 months	130	88·574	15·00	124	89·600	13·01	−0·270	−2·319 to 1·780	0·796
12 months	125	89·999	14·44	118	88·683	13·17	1·558	−0·535 to 3·650	0·145
18 months	113	89·860	14·35	107	88·882	12·32	−0·210	−2·386 to 1·967	0·850
Stroke Impact Scale (SIS), Mobility									
Baseline	150	88·817	15·43	144	88·611	15·85			
6 months	132	88·731	15·82	126	90·139	12·54	−1·345	−3·614 to 0·923	0·245
12 months	124	90·605	13·34	118	89·258	14·90	0·345	−1·985 to 2·675	0·772
18 months	115	90·283	14·54	109	89·495	13·10	−0·871	−3·273 to 1·530	0·477
Stroke Impact Scale (SIS), Communication									
Baseline	151	91·533	9·36	146	92·466	12·49			
6 months	130	90·412	10·46	127	91·535	10·48	−0·974	−2·934 to 0·985	0·330
12 months	125	91·800	9·32	118	91·707	9·64	0·680	−1·330 to 2·689	0·507
18 months	114	91·416	9·51	107	91·923	10·06	−0·302	−2·394 to 1·790	0·777
Stroke Impact Scale (SIS), Emotion									
Baseline	148	84·347	14·13	146	84·684	14·50			
6 months	132	83·354	14·61	128	84·787	13·57	−1·716	−4·579 to 1·147	0·240
12 months	122	83·789	15·33	118	85·005	13·21	−0·907	−3·874 to 2·060	0·549
18 months	113	84·857	14·52	106	86·609	13·52	−1·660	−4·746 to 1·425	0·292
Stroke Impact Scale (SIS), Memory and thinking									
Baseline	150	86·750	12·48	146	87·008	12·76			
6 months	131	86·045	12·81	128	87·793	10·58	−2·254	−4·517 to 0·008	0·051
12 months	126	87·302	12·91	120	85·964	12·13	1·201	−1·116 to 3·517	0·310
18 months	113	87·694	12·61	107	88·522	12·59	−1·753	−4·178 to 0·673	0·157
Stroke Impact Scale (SIS), Participation/Role function									
Baseline	134	79·913	19·26	134	79·809	20·71			
6 months	119	85·061	16·94	118	85·287	17·37	−1·038	−4·693 to 2·618	0·578
12 months	113	84·464	17·97	111	83·358	19·94	1·219	−2·532 to 4·970	0·524
18 months	104	85·710	17·78	102	86·002	16·20	−1·471	−5·364 to 2·420	0·459

a The group × time interactions are relative to baseline, with the control group as the reference group for these between group differences. The between group differences are adjusted for age, dependency level (mRS at inclusion), hospital site, sex and the admission National Institute of Health Stroke Scale (NIHSS) score as a measure of stroke severity.

The analyses did not identify any significant differences between the groups for any secondary outcome measures as displayed in Tables 2 and in the Supplementary Material (Supplementary Table S1).

The sensitivity analysis substantiated the results, confirming their robustness (Supplementary Table S2).

The subgroup analysis displayed in the Supplementary Material (Supplementary Table S3) showed no effect for any of the subgroups despite a significant difference in favour of the intervention group at 12 months for participants recruited at Hospital 3 with an estimated difference of 0·40 (95% CI: 0·003–0·804) points (p = 0·048) and at 18 months for participants recruited at Hospital 4 with an estimated difference of 0·711 (95% CI: 0·175–1·247) points (p = 0·009).

The occurrence of serious adverse events was equally distributed between the intervention and control groups (Table 3). In total, eight participants died during the 18 months follow-up period, whereof three in the intervention group and five in the control group.

**Table 3:** Serious adverse events (SAE)^a^ during follow-up in the intervention and control group.

	Intervention (n = 152)	Control (n = 149)
Adverse events, n (%)		
Death	3 (2·0)	5 (3·3)
Cerebral infarction	5 (3·3)	6 (4·0)
Cerebral hemorrhage	3 (2·0)	1 (0·7)
Transitory Ischemic Attack (TIA)	2 (1·3)	1 (0·7)
Myocardial infarction	1 (0·7)	0 (0·0)
Atrial fibrillation	3 (2·0)	6 (4·0)
Heart failure	2 (1·3)	3 (2·0)
Fall with fracture	9 (5·9)	3 (2·0)
Fall without fracture	4 (2·6)	8 (5·4)

a SAEs were defined as death or hospitalisation due to one of the events listed in the table.

A total of 96 (63·2%) participants in the intervention group achieved 75% adherence to the intervention (Supplementary Table S4). Eight participants discontinued the intervention before the first meeting, while two participants attended all 18 meetings. The mean number of meetings per participant randomised to the intervention group was 12·3 (SD 5·1).

A total of 1540 out of 2584 potential evaluations of goal achievement and 1519 evaluations of adherence to the action plans were available in the dataset. The results showed a mean goal achievement of 2·9 (SD 1·3) points (which corresponds to neither agree nor disagree) and adherence to the action plan of 3·7 (SD 0·7) points (which corresponds to partly agree). See Supplementary Materials (Supplementary Figure S2).

## Discussion

This trial showed that an 18-month multimodal personalized intervention delivered by a dedicated stroke-coordinator was not superior to the standard care received by the control group in preventing dependency in activities of daily living in people with mild strokes.

The modified Rankin Scale was chosen as the primary outcome. In our power calculations, a reduction of 0·4 points was considered as a clinically significant reduction, however, both groups deteriorated about 0·1 points from baseline to 18 months, with no significant differences observed between them. Even though the target group for this intervention was home-dwelling stroke survivors with moderate impairments, the study sample was mainly characterized by mild strokes according to NIHSS. More than 85% of the participants were classified as independent with a mRS score between zero and two at inclusion, which indicates a low risk of functional decline independent of group allocation.^24^ As only 18% (301 out of 1656 participants) were included, it is likely that we failed to recruit the frailest individuals - those who might have benefitted most from the intervention.^25^ According to the recruitment staff, the patients with more severe strokes and a larger number of comorbidities, especially those with cognitive decline, were more likely to decline participation. Additionally, patients who were discharged to in-patient rehabilitation were less likely to attend the out-patient clinic within the inclusion window (two to four months post stroke) and were for that reason not considered for inclusion.

Furthermore, the results demonstrated no differences between the groups for any secondary outcomes or in the sensitivity analyses. Nevertheless, for the TMT-B test, the estimated reduction in time from baseline to 18-month follow-up was 14·08 s greater in the intervention group compared to the control group, while the corresponding change was 16·44 s in the sensitivity analysis. Despite the lack of a statistically significant difference one may argue that a 10% reduction in time spent on TMT-B is clinically significant. TMT-B is a measure of executive function, which is one of the cognitive domains that has shown to benefit from motor- and cardiorespiratory training.^26^ Even though this trial was not powered to detect a change on TMT-B, we cannot preclude a beneficial effect of the multimodal intervention on cognition, which also is in line with findings from the FINGER study aiming to prevent cognitive decline in older adults.^27^

Another finding from the secondary analysis was the significant differences between the groups at 12 months for participants recruited at Hospital 3 and at 18 months for participants recruited at Hospital 4 is indicating that there might be local differences in the implementation of the intervention that need to be further explored in the process evaluation.

Serious adverse events were recorded prospectively from the participants during follow-up and quality assured retrospectively from the patient records. An intervention aiming to increase activity levels in stroke survivors has the potential to also increase the risk of serious adverse events like falls and new cardiovascular events. It seems like more participants in the intervention group (nine versus three participants) were hospitalized due to falls with fracture, while more participants in the control group (eight versus four participants) were hospitalized due to falls without fracture. We consider these differences to be random as whether hospitalisation due to a fall is with or without a fracture is most likely depending on features by the patient and not by the intervention. Nevertheless, fall is a serious adverse event in this population and fall prevention should be included as part of future multimodal interventions. Unfortunately, we were not able to reliably collect adverse events (AEs), as this would have required continuous monitoring of both groups. Consequently, we cannot exclude the possibility that there were differences between the groups in for example falls that did not result in hospitalisation.

It has been argued that the LAST-long intervention should be considered a complex intervention according to the latest update from the UK Medical Research Council.^18^ The advantage of such an intervention is the multiple components and its flexibility close to real life. However, the disadvantage is the challenges related to the great variability and lack of standardization, making it hard to conclude on which elements are the most effective parts of the intervention. In addition to risk factor management, the key ingredients of the LAST-long intervention was motivational interviewing, shared decision making and goal setting, and coordination and support to enter recommended services. Still, it is hard to know whether it was the support provided by the stroke coordinators or poor adherence to the treatment plan caused by characteristics by the participants that contributed to the neutral findings. In addition to the previously mentioned multimodal trials, the LAST-long intervention has features consistent with the role of patient navigation investigated in two long-term rehabilitation trials.^28^^,^^29^ These trials also revealed neutral results on clinical outcomes and cost-effectiveness, indicating that the coordination or the navigation might be the less effective part of the intervention. Hence, future research needs to develop and explore new methods on how to improve adherence to the intervention even more, like for example regular reminders by digital applications on smart phones or smart watches.

Another challenge during this trial was the COVID-19 pandemic. The lockdown and restrictions related to social distancing were a significant barrier against trial recruitment and adherence to the intervention. Our results showed that 96 (63·2%) of the participants in the intervention group reached the level of good adherence, i.e., attended at least 14 out of 18 meetings. Furthermore, we were aiming for at least 50% of the meetings to be in-person and achieved 51·8%, which was regarded as very good, given the two years with a pandemic during this trial. Thus, the intervention should be regarded as feasible, although withdrawal from the intervention exceeded 10% indicates barriers to participate for some individuals.

Regarding goal achievement and adherence to the treatment plan, participants on average reported neutral response to the statement “I achieved my goals”, while they partly agreed with the statement “I adhered to the action plan”. Despite the use of motivating interviewing and the shared decision-making process, one possible explanation of this finding is that the goals set may have been somewhat unrealistic, making them difficult to achieve even though the participants partly adhered to the action plan. This is an important finding that should be considered when goal setting is applied in future research and clinical practice.

This is the largest trial of its kind, assessing the impact of an 18-month coordinator-led personalized, multidomain approach to long-term follow-up after stroke. The randomised controlled trial with repeated assessments performed by well-trained and experienced assessors applying a comprehensive test-battery at 6-, 12-, and 18-month follow-up, blinded to group allocation is also a major strength. The fact that the intervention was delivered by a dedicated community-based stroke-coordinator with proximity to all resources and support functions within the municipality must also be considered beneficial. Emphasizing the individualized intervention which involved patients setting their own goals was considered crucial.

When comparing the LAST-long sample with the total Norwegian stroke population from 2024, we found that our sample consisted of slightly more women, with younger age and less severe stroke. Given that patients with mRS score of 5 at three months follow up were excluded from participation, we find our sample highly representative to the majority of the Norwegian stroke population with mild strokes.

The method of utilizing distinct personnel for participant screening, intervention delivery, and testing the participants restricted bias in the dataset. A final strength is the use of mixed model that can handle missing data under the presumption that data is missing-at-random. For the primary outcome, 16 participants in the intervention group and 17 participants in the control group were missing at 18-month follow-up. The reasons for missing data were most likely related to other observed characteristics by the participants and not to the intervention itself, thus, the presumption for mixed model was most likely fulfilled.

Some limitations of this study need to be acknowledged. Even though social function was one of the domains that was addressed in the checklist, no screening tool on social function was included. It is also a limitation that the dominant/non-dominant arm was not registered when grip strength was measured. Furthermore, we could not be sure that the impairments required to be included, as defined by our inclusion criteria, actually were caused by the stroke when a pre-stroke measure was lacking. However, this limitation applies to most stroke trials.

Unfortunately, this trial was not powered for sex specific analysis. A meta-analysis investigating sex differences in long-term outcomes found that the risk of recurrent stroke, dependency, and mortality was less in men at 1-year follow-up, while there were no differences in health-related quality of life or depression at any time point.^30^ Even though, our subgroup analysis showed no differences between the groups for female and male participants, we cannot preclude that men and women respond differently to coordinated support and future trials should be designed to include prespecified sex specific analyses.

Even though very few trials have investigated the effects of lifestyle interventions lasting for 18 months or more, it is well known that it might require additional time to measure the effect of such interventions. Therefore, an even longer follow-up period investigating the effect of multimodal interventions on hard outcomes, like cardiovascular events and deaths in a 5- or 10-year perspective is required.

The Norwegian health care system is known to be of very high quality, providing governmental funded health care and services to all inhabitants regardless of ethnicity or socioeconomic background. Thus, the standard care provided to the control group might also encompasses support from the primary healthcare system for those in need. This reality makes it hard to improve the services even more and provide evidence for long-term follow-up by a dedicated stroke coordinator. Accordingly, our results do not support the implementation of an 18-month coordinator-led multimodal intervention into clinical practice in people with mild strokes.

Even though the intervention was considered to be feasible and safe, an 18-month coordinator-led multimodal intervention program was not superior to the standard care received by the control group in preventing dependency or decline in ADL-function, cognitive function, physical function, physical activity, patient reported outcome measures and secondary prevention outcome measures in people with mild stroke. These findings should be considered as an important part of the evidence for long-term follow-up after stroke. The process evaluation will inform future studies on how to reach the frail part of the stroke population that might benefit most from a coordinator-led multimodal intervention and how to improve adherence to the intervention even more.

## Contributors

TA was the principal investigator and project manager of the LAST-long trial. TA and SRL wrote the original draft of this manuscript. EB, ØD, HE, AH, HI–H, BI, SL, GL, YS, TS, IS and BT critically reviewed and commented on the paper. TA, IS and BT conceptualized the trial. TA, ØD, AH, BI, IS and BT developed the study design. TA was responsible for the funding acquisition. TA, TS and BT took part in supervision. HE, HI–H, BI, YS and BT provided access to the recruitment of participant and coordinated the research activity at the participating hospitals. ØD provided access to follow-up of participants in the municipality. AH coordinated the intervention. SRL, EB, AH, GL collected data. TA and SRL curated the data. TA and SRL verified the data. TA performed the analyses presented in Tables 1, 3, S4, and some of the variables included in Tables S1 and S2. SRL conducted the analyses for Table 2, Table S3, and most of the variables included in Tables S1 and S2. GL performed the analysis for Figure S2. SL, the study statistician, supervised and reviewed all analyses. SRL visualized the results. TA, SRL, EB, ØD, HE, AH, HI–H, BI, SL, GL, YS, TS, IS and BT interpreted the results. All authors had full access to the data. All authors approved the final version of the manuscript and were responsible for the decision to submit the manuscript for publication.

## Data sharing statement

In line with the ethical approval restrictions apply to the availability of data until January 2033. However, after that date, anonymous individual data can be available upon request to the corresponding author.

## Declaration of interests

All authors declare no competing interests.
