# Supplementary material for: A multimodal individualized long-term intervention to prevent functional decline after stroke (LAST-long): a single blinded randomised controlled trial
Source: Lancet Reg Health Eur. 2025 Nov 13;61:101531. doi: 10.1016/j.lanepe.2025.101531 (PMC12662110; doi:10.1016/j.lanepe.2025.101531)
Supplement: Multimedia component 1 [file mmc1.pdf]

Supplementary Material

Table of contents

Content	Page
Table S1	2
Table S2	3
Table S3	6
Table S4	6
Figure S1	7
Figure S2	8
Sample size estimation	8
Ethical approval	9

Table S1. Secondary prevention outcomes

	Intervention group (n=152)			Control group (n=149)			Difference (Group x Time)*		
	n	Mean	SD	n	Mean	SD	Estimate	95% CI	p-value
**Physical activity, mean number of steps per day**									
Baseline	108	5474·5	3243·9	100	5768·8	3883·2			
6 months	86	5496·1	2821·6	72	6437·6	3494·5	-257·86	-961·63 to 445·90	0·47
12 months	73	5829·0	3401·2	69	6021·7	3513·1	265·78	-466·99 to 998·55	0·48
18 months	63	5782·5	3751·7	49	6083·3	3545·8	-138·07	-961·18 to 685·05	0·74
**Systolic blood pressure, mmHg**									
Baseline	151	140·30	16·45	146	140·45	18·17			
6 months	123	139·11	19·84	120	138·57	20·29	1·15	-2·98 to 5·27	0·59
12 months	114	139·03	21·35	111	137·69	20·22	2·49	-1·78 to 6·77	0·25
18 months	107	135·60	17·43	98	136·91	19·55	-2·08	-6·54 to 2·37	0·36
**Body mass index (BMI), kg/m^2**									
Baseline	140	27·12	5·90	137	26·37	4·26			
6 months	117	27·04	4·58	118	26·29	4·55	-0·08	-0·76 to 0·59	0·81
12 months	109	26·59	4·40	103	26·33	3·86	-0·35	-1·06 to 0·35	0·33
18 months	106	26·84	4·66	97	25·91	3·99	-0·18	-0·90 to 0·54	0·62
**Total cholesterol, mmol/L**									
Baseline	140	3·92	1·52	134	4·13	1·60			
6 months	102	3·89	1·07	96	4·35	1·98	-0·30	-0·62 to 0·026	0·07
12 months	97	3·83	0·94	89	3·95	0·95	-0·01	-0·36 to 0·32	0·95
18 months	91	3·86	0·93	84	3·84	0·91	0·09	-0·25 to 0·44	0·60
**Low-density lipoprotein (LDL), mmol/L**									
Baseline	140	2·06	0·90	133	2·22	0·85			
6 months	101	2·10	0·93	98	2·26	0·91	0·02	-0·15 to 0·18	0·84
12 months	96	2·03	0·86	90	2·14	0·85	0·04	-0·13 to 0·21	0·63
18 months	91	2·05	0·78	84	2·06	0·77	0·09	-0·08 to 0·27	0·31
**Glycated haemoglobin (HbA1c), mmol/mol**									
Baseline	136	41·24	8·64	132	41·28	7·83			
6 months	98	41·43	10·69	97	40·40	6·87	1·37	-0·22 to 2·95	0·091
12 months	98	40·43	9·73	89	41·81	8·92	-0·91	-2·53 to 0·70	0·27
18 months	87	42·64	11·04	84	41·43	7·23	1·80	0·13 to 3·47	0·034
**C-reactive protein (CRP), mg/L**									
Baseline	123	2·96	6·01	120	3·63	10·90			
6 months	94	3·55	6·25	88	3·82	6·00	-0·07	-1·98 to 1·83	0·94
12 months	92	3·12	4·31	88	3·95	6·19	-0·61	-2·52 to 1·30	0·53
18 months	88	3·80	5·43	81	3·71	5·74	0·27	-1·70 to 2·24	0·79

*The group x time interactions are relative to baseline, with the control group as the reference group for these between group differences. The between group differences are adjusted for age, dependency level (mRS at inclusion), hospital site, sex and the admission National Institute of Health Stroke Scale (NIHSS) score as a measure of stroke severity.

Table S2. Sensitivity analysis. Primary and secondary outcomes

	Intervention group (n=152)			Control gruop (n=149)			Difference (Group x Time)*		
	n	Mean	SD	n	Mean	SD	Estimate	95% CI	p-value
**modified Rankin Scale (mRS)**									
Baseline	96	1·59	0·89	149	1·66	0·86			
6-month	95	1·51	0·82	143	1·64	1·00	-0·09	-0·30 to 0·11	0·38
12-month	93	1·55	0·89	133	1·68	1·03	-0·12	-0·32 to 0·09	0·27
18-month	93	1·54	0·89	132	1·78	1·27	-0·13	-0·34 to 0·08	0·23
**Barthel Index**									
Baseline	95	96·37	9·15	147	97·24	5·89			
6-month	90	97·33	5·72	137	96·46	6·45	0·66	-0·86 to 2·19	0·39
12-month	92	96·25	7·69	122	96·11	8·44	0·34	-1·21 to 1·88	0·67
18-month	90	95·50	10·80	118	96·73	7·26	-1·23	-2·79 to 0·34	0·12
**Nottingham I-ADL**									
Baseline	96	53·45	12·13	148	54·16	11·50			
6-month	87	57·25	9·76	134	56·44	10·60	0·51	-1·40 to 2·42	0·60
12-month	88	57·28	11·85	118	57·16	11·30	0·94	-1·00 to 2·89	0·34
18-month	87	57·60	12·24	113	55·50	11·44	1·84	-0·13 to 3·80	0·067
GDS									
Baseline	96	1·97	0·81	149	2·17	0·95			
6-month	92	1·95	0·83	132	2·16	1·01	-0·05	-0·26 to 0·16	0·65
12-month	89	1·83	0·97	120	2·06	1·0	-0·13	-0·34 to 0·09	0·24
18-month	87	1·89	1·04	110	2·02	1·04	0·03	-0·19 to 0·25	0·81
**Montreal Cognitive Assessment (MoCA)**									
Baseline	95	24·57	3·73	149	24·36	3·67			
6-month	84	25·21	3·26	119	24·92	3·83	-0·27	-0·96 to 0·42	0·44
12-month	81	25·74	3·82	109	25·35	3·93	0·22	-0·48 to 0·93	0·54
18-month	78	26·36	2·99	97	25·16	4·33	0·65	-0·07 to 1·38	0·078
**Trail Making Test A (TMT-A)**									
Baseline	95	53·25	29·70	146	59·23	38·68			
6-month	85	48·03	22·75	115	54·87	35·47	-1·21	-8·17 to 5·76	0·73
12-month	80	51·19	28·85	107	55·60	36·95	-0·82	-7·97 to 6·32	0·82
18-month	77	46·57	27·21	95	58·07	45·43	-4·39	-11·78 to 2·99	0·24
**Trail Making Test B (TMT-B)**									
Baseline	88	137·84	75·25	127	139·85	82·86			
6-month	81	143·47	82·41	107	154·41	94·71	-4·06	-20·55 to 12·42	0·63
12-month	76	140·20	82·52	95	144·54	84·11	-5·09	-22·17 to 12·00	0·56
18-month	72	126·70	79·90	87	149·86	90·59	-16·44	-34·04 to 1·16	0·067
**Short Physical Performance Battery (SPPB)**									
Baseline	95	10·05	2·62	147	9·88	2·59			
6 months	83	9·83	2·41	120	9·33	2·60	0·45	-0·03 to 0·93	0·064
12 months	82	9·76	2·13	109	9·29	2·79	0·11	-0·38 to 0·60	0·66
18 months	78	9·71	2·52	97	9·53	2·52	-0·21	-0·72 to 0·30	0·42
**Six Minute Walk Test (6MWT)**									
Baseline	81	377·71	111·94	123	361·39	123·36			
6 months	75	378·20	126·80	110	365·0	134·93	0·32	-19·90 to 18·54	0·97
12 months	78	388·39	132·50	102	361·79	139·35	11·89	-6·42 to 30·19	0·20
18 months	71	405·83	122·82	90	366·41	133·63	11·04	-7·90 to 29·98	0·25
**Gripstrength right**									
Baseline	95	30·12	13·68	145	28·85	11·86			
6 months	78	30·86	14·48	117	28·85	12·09	0·42	-1·12 to 1·95	0·59
12 months	81	30·56	13·83	110	28·99	12·78	0·32	-1·22 to 1·85	0·68
18 months	76	30·92	13·76	96	29·25	13·07	0·71	-0·89 to 2·30	0·39
**Gripstrength left**									
Baseline	95	28·35	12·30	144	25·88	12·37			
6 months	78	29·13	13·23	117	25·48	12·29	1·10	-0·35 to 2·56	0·14
12 months	82	29·55	12·95	110	25·78	12·88	1·27	-0·18 to 2·72	0·086
18 months	75	30·13	12·36	96	25·67	13·36	1·49	-0·03 to 3·01	0·055
**Fatigue Severity Scale, seven item version (FSS-7)**									
Baseline	96	3·61	1·95	148	3·43	2·09			
6 months	91	3·91	1·75	132	3·79	1·87	-0·05	-0·44 to 0·34	0·80
12 months	91	3·54	1·64	119	3·41	1·86	0·02	-0·38 to 0·42	0·93
18 months	84	3·86	1·82	108	3·39	1·91	0·25	-0·17 to 0·66	0·24
**Hospital Anxiety and Depression Scale, total (HADS)**									
Baseline	96	6·75	6·23	148	5·90	5·77			
6 months	91	8·44	5·45	132	7·99	6·20	-0·22	-1·36 to 0·93	0·71

12 months	90	8·11	6·11	119	8·07	5·79	-0·50	-1·67 to 0·67	0·40
18 months	84	8·36	6·13	107	7·15	6·01	0·33	-0·88 to 1·54	0·60
**Hospital Anxiety and Depression Scale, Anxiety (HADS-A)**									
Baseline	96	3·64	3·58	148	3·30	3·73			
6 months	91	4·34	3·23	132	3·99	3·79	0·09	-0·62 to 0·81	0·80
12 months	90	4·11	3·47	119	4·15	3·70	-0·13	-0·86 to 0·60	0·72
18 months	84	4·36	3·46	108	3·65	3·73	0·34	-0·42 to 1·10	0·38
**Hospital Anxiety and Depression Scale, Depression (HADS-D)**									
Baseline	96	3·12	3·25	148	2·61	2·63			
6 months	91	4·10	3·18	132	4·00	3·32	-0·28	-·092 to 0·36	0·39
12 months	90	4·00	3·45	119	3·92	2·94	-0·34	-0·99 to 0·32	0·32
18 months	84	4·00	3·40	107	3·47	3·15	0·04	-0·64 to 0·72	0·90
**EQ-5D-5L index**									
Baseline	96	0·84	0·15	149	0·84	0·16			
6 months	89	0·81	0·15	132	0·82	0·17	-0·05	-0·05 to 0·02	0·38
12 months	90	0·85	0·15	122	0·83	0·17	0·02	-0·02 to 0·05	0·27
18 months	87	0·85	0·14	113	0·85	0·02	-0·04	-0·04 to 0·03	0·82
**EQ-5D-5L VAS**									
Baseline	95	69·92	14·14	148	65·78	19·59			
6 months	88	67·02	16·21	131	67·68	18·33	-2·93	-6·95 to 1·08	0·15
12 months	86	72·00	16·14	118	68·48	18·03	1·19	-2·92 to 5·30	0·57
18 months	83	69·45	16·64	110	70·41	18·89	-2·53	-6·73 to 1·68	0·24
**Stroke Impact Scale (SIS), over all recovery**									
Baseline	94	73·40	18·01	147	70·61	21·18			
6 months	90	74·77	18·62	131	70·47	24·28	1·48	-3·16 to 6·12	0·53
12 months	89	75·02	21·59	119	72·05	24·36	1·57	-3·15 to 6·30	0·51
18 months	82	75·66	17·07	109	72·61	22·82	0·09	-4·81 to 4·99	0·97
**Stroke Impact Scale (SIS), domain strength**									
Baseline	94	80·12	20·76	144	80·82	23·57			
6 months	89	79·07	23·22	128	79·25	23·45	1·40	-2·80 to 5·60	0·51
12 months	89	80·90	21·62	119	81·67	22·32	0·58	-3·68 to 4·84	0·79
18 months	82	81·17	20·66	107	81·13	22·45	0·78	-3·64 to 5·20	0·73
**Stroke Impact Scale (SIS), domain hand function**									
Baseline	95	85·58	21·72	145	85·03	22·62			
6 months	88	85·97	22·20	126	86·63	22·21	-0·58	-4·01 to 2·85	0·74
12 months	87	86·44	21·86	118	86·10	24·38	0·19	-3·30 to 3·67	0·92
18 months	83	89·16	19·11	108	86·44	24·50	0·59	-2·98 to 4·15	0·75
**Stroke Impact Scale (SIS), domain ADL / iADL**									
Baseline	95	89·56	13·46	144	88·53	13·50			
6 months	87	90·40	13·19	124	89·60	13·01	0·44	-1·67 to 2·55	0·68
12 months	89	90·54	14·75	118	88·68	13·17	0·96	-1·16 to 3·08	0·38
18 months	81	91·28	12·87	107	88·88	12·32	0·27	-1·93 to 2·47	0·81
**Stroke Impact Scale (SIS), domain mobility**									
Baseline	94	89·81	14·80	144	88·61	15·85			
6 months	89	88·93	16·50	126	90·14	12·54	-1·83	-4·31 to 0·64	0·15
12 months	88	90·74	13·69	118	89·26	14·90	0·18	-2·33 to 2·69	0·89
18 months	83	91·81	12·87	109	89·50	13·10	-0·17	-2·75 to 2·40	0·90
**Stroke Impact Scale (SIS), domain communication**									
Baseline	95	91·39	9·61	146	92·47	12·49			
6 months	87	90·56	9·76	127	91·54	10·48	-0·59	-2·74 to 1·57	0·59
12 months	89	91·65	9·10	118	91·71	9·64	0·71	-1·46 to 2·88	0·52
18 months	82	91·77	8·94	107	91·92	10·06	0·25	-2·01 to 2·50	0·83
**Stroke Impact Scale (SIS), domain emotion**									
Baseline	92	85·60	13·93	146	84·68	14·50			
6 months	89	82·49	14·77	128	84·79	13·57	-2·40	-5·55 to 0·76	0·14
12 months	88	83·84	15·10	118	85·01	13·21	-1·21	-4·42 to 2·00	0·46
18 months	82	84·89	14·85	106	86·61	13·52	-2·01	-5·34 to 1·32	0·24
**Stroke Impact Scale (SIS), domain memory and thinking**									
Baseline	94	86·97	11·11	146	87·01	12·76			
6 months	88	86·44	12·45	128	87·79	10·58	-1·58	-4·09 to 0·93	0·22
12 months	90	87·71	12·16	120	85·96	12·13	1·71	-0·81 to 4·24	0·18
18 months	82	88·42	11·68	107	88·52	12·59	-0·83	-3·46 to 1·80	0·54
**Stroke Impact Scale (SIS), domain participation / role function**									
Baseline	82	82·28	17·02	134	79·81	20·71			
6 months	81	86·32	14·78	118	85·29	17·37	-0·73	-4·63 to 3·18	0·72
12 months	79	85·34	15·49	111	83·36	19·94	1·13	-2·85 to 5·10	0·58
18 months	74	87·80	15·18	102	86·00	16·20	0·47	-3·64 to 4·58	0·82
**Physical activity, mean number of steps per day**									
Baseline	69	5722·29	3074·04	100	5762·79	3883·15			
6 months	64	5658·58	2825·12	72	6437·63	3494·48	-187·77	-949·06 to 573·52	0·63
12 months	56	5796·21	3241·16	69	6021·68	3513·05	247·97	-540·36 to 1036·31	0·54

18 months	44	5945·16	3405·90	49	6083·35	3545·80	-52·26	-949·30 to 844·77	0·91
**Systolic blood pressure, mmHg**									
Baseline	95	140.08	16.60	146	140.45	18·17			
6 months	83	137·41	19·87	120	138·57	20·29	0·90	-3·67 to 5·47	0·70
12 months	82	138·34	20·05	111	137·69	20·22	1·72	-2·92 to 6·37	0·47
18 months	81	134·12	17·21	98	136·91	19·55	-3·22	-8·0 to 1·56	0·19
**Body mass index (BMI), kg/m^2**									
Baseline	89	27·29	6·41	137	26·37	4·26			
6 months	80	27·42	4·56	118	26·29	4·55	0·06	-0·74 to 0·85	0·89
12 months	79	26·89	4·39	103	26·33	3·86	-0·31	-1·13 to 0·51	0·46
18 months	78	26·97	4·79	97	25·91	3·99	-0·17	-0·99 to 0·66	0·69
**Total cholesterol, mmol/L**									
Baseline	91	3·90	1·06	134	4·13	1·60			
6 months	76	3·88	1·04	96	4·35	1·98	-0·24	-0·57 to 0·092	0·16
12 months	70	3·85	1·00	89	3·95	0·95	0·04	-0·031 to 0·38	0·83
18 months	69	3·93	0·96	84	3·84	0·91	0·19	-0·16 to 0·54	0·30
**Low-density lipoprotein (LDL), mmol/L**									
Baseline	91	2·10	0·91	133	2·22	0·85			
6 months	75	2·09	0·88	98	2·26	0·91	-0·00	-0·18 to 0·18	0·99
12 months	69	2·06	0·90	90	2·14	0·85	0·03	-0·16 to 0·21	0·76
18 months	69	2·08	0·74	84	2·06	0·77	0·07	-0·11 to 0·26	0·44
**Glycated haemoglobin (HbA1c), mmol/mol**									
Baseline	88	40·11	7·06	132	41·28	7·83			
6 months	74	41·23	10·75	97	40·40	6·87	1·36	-0·35 to 3·08	0·12
12 months	71	39·43	9·51	89	41·81	8·92	-1·47	-3·22 to 0·28	0·10
18 months	65	40·98	7·92	84	41·43	7·23	1·12	-0·68 to 2·92	0·22
**C-reactive protein (CRP), mg/L**									
Baseline	79	2·96	5·47	120	3·63	10·90			
6 months	72	3·56	6·57	88	3·82	6·00	-0·10	-2·19 to 2·00	0·93
12 months	68	3·09	3·97	88	3·95	6·19	-0·56	-2·68 to 1·56	0·61
18 months	67	4·20	6·07	81	3·71	5·74	0·60	-1·57 to 2·78	0·59

*The group x time interactions are relative to baseline, with the control group as the reference group for these between group differences. The between group differences are adjusted for age, dependency level (mRS at inclusion), hospital site, sex and the admission National Institute of Health Stroke Scale (NIHSS) score as a measure of stroke severity.

Table S3. Subgroup analyses with mRS as the outcome variable

	Difference (Group x Time)*			Difference (Group x Time)*		
	Estimate	95%CI	p-value	Estimate	95%CI	p-value
	Female			Male		
6 months	-0·003	-0·26 to 0·26	0·98	0·05	-0·21 to 0·31	0·70
12 months	-0·07	-0·34 to 0·21	0·63	0·11	-0·16 to 0·37	0·42
18 months	0·03	-0·25 to 0·30	0·85	0·05	-0·21 to 0·31	0·71
	NIHSS at admission < 8			NIHSS at admission ≥ 8		
6 months	0·08	-0·10 to 0·25	0·40	-0·37	-0·94 to 0·20	0·20
12 months	0·10	-0·08 to 0·28	0·29	-0·52	-1·11 to 0·06	0·08
18 months	0·04	-0·14 to 0·23	0·65	-0·06	-0·63 to 0·51	0·84
	Age at inclusion ≥ 80 years			Age at inclusion < 80 years		
6 months	-0·03	-0·21 to 0·15	0·73	0·14	-0·27 to 0·55	0·51
12 months	0·09	-0·09 to 0·27	0·33	-0·26	-0·70 to 0·18	0·25
18 months	-0·002	-0·18 to 0·18	0·98	0·09	-0·35 to 0·52	0·69
	Hospital 1			Hospital 2		
6 months	0·07	-0·24 to 0·37	0·66	-0·08	-0·32 to 0·17	0·54
12 months	-0·05	-0·35 to 0·26	0·77	-0·11	-0·36 to 0·14	0·39
18 months	-0·05	-0·37 to 0·26	0·75	0·04	-0·22 to 0·29	0·77
	Hospital 3			Hospital 4		
6 months	0·17	-0·22 to 0·56	0·40	0·461	-0·08 to 0·997	0·09
12 months	0·40	0·003 to 0·80	0·048	0·248	-0·59 to 1·084	0·56
18 months	0·14	-0·26 to 0·53	0·50	0·711	0·18 to 1·247	0·009

*The group x time interactions are relative to baseline, with the control group as the reference group for these between group differences. The between group differences are adjusted for age, dependency level (mRS at inclusion), hospital site, sex and the admission National Institute of Health Stroke Scale (NIHSS) score as a measure of stroke severity.

Table S4. Adherence to the intervention (n=152)

	**Attended meetings**
Meeting number	n (%)
No 1	139 (91·4)
No 2	120 (78·9)
No 3	119 (78·3)
No 4	112 (73·7)
No 5	111 (73·0)
No 6	107 (70·4)
No 7	99 (65·1)
No 8	100 (65·8)
No 9	106 (69·7)
No 10	97 (63·8)
No 11	93 (61·2)
No 12	96 (63·2)
No 13	94 (61·8)
No 14	94 (61·8)
No 15	90 (59·2)
No 16	94 (61·8)
No 17	90 (59·2)
No 18	105 (69·1)
Total	1866 (68·2)
Face-to-face	977 (52·3)

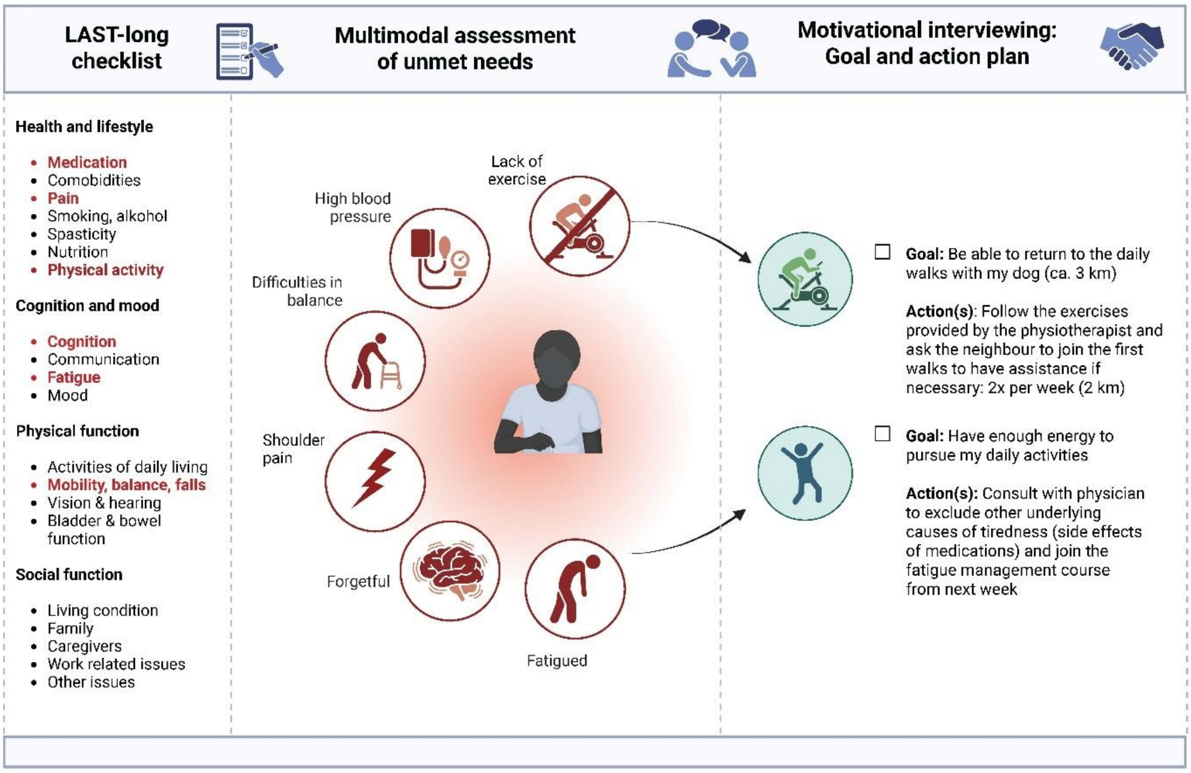

Figure S1. The LAST-long study intervention: An example

The Figure shows an applied example of the LAST-long study intervention. The 4 different domains and their sub-domains covered by the LAST-long checklist are presented in the left column. Note: Using the LAST-long checklist, each of the 20 subdomains was assessed as either relevant or not relevant for the individual's health and life after stroke at the time of assessment. Based on the assessment, motivational interviewing helped determine individuals' goals, which were subsequently addressed in a treatment plan. Maximum domain scores that could have been reached varied across the domains: Health and lifestyle: 7; Cognition and mood: 4; Physical function: 4; and Social function: 5.

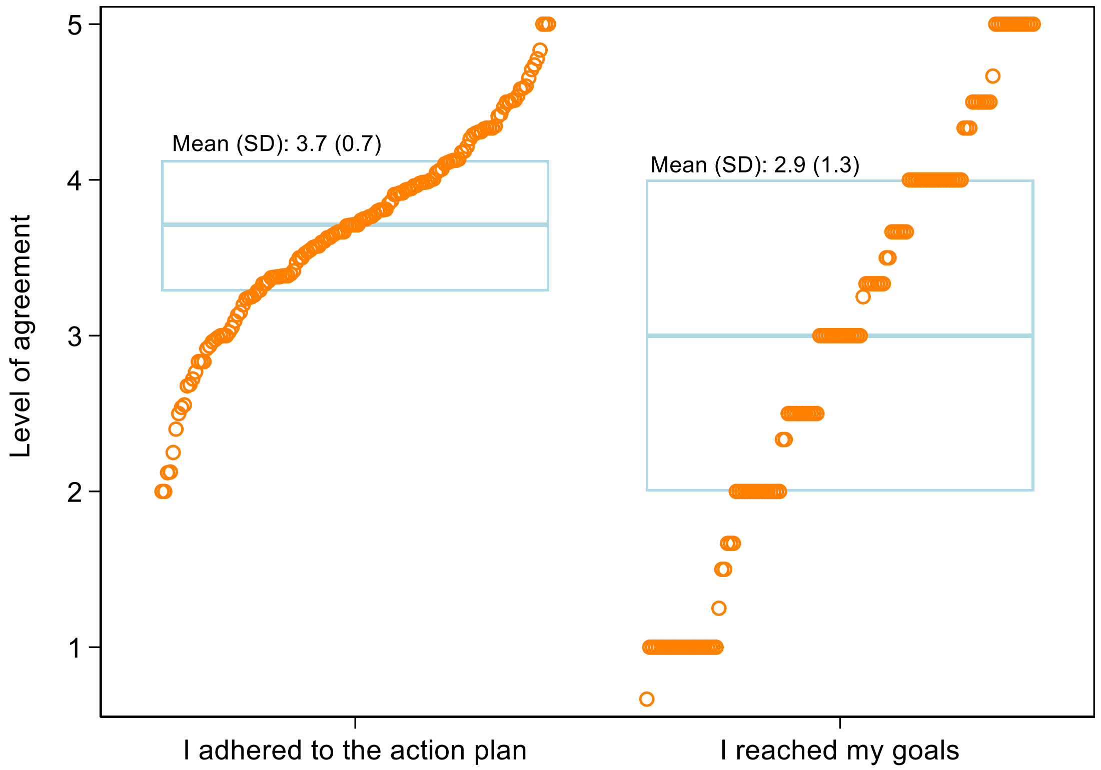

Figure S2. Adherence to the intervention.

Box plots combined with the detail of quantile plots. 1=Fully disagree, 2=Partly disagree, 3=Neither agree nor disagree, 4= partly agree, 5= fully agree

Analysis

Sample size estimation

In the original version of the protocol at Clinicaltrials.gov the sample size estimation was based on an 80 % power at a significance level 0.05 for a mean difference of 0.4 and an average standard deviation of 1.46, giving a total of 210x2=420 participants. We expected about 15% drop-out and the total number was adjusted to 490 participants. However, in June 2021 it became clear that obtaining a sample size of 490 would be unrealistic mainly because of the pandemic and we decided to reduce the power and re-estimate the sample size. A sample size of approximately 150x2=300 seems realistic at this point in time. The sample size calculation method closest to the mixed model analysis available in the sample size software NCSS 2020, is ANCOVA. Based on the LAST study (Askim et al., BMJ Open 2023), we estimated the correlation between mRS at baseline and 18 months to be R=0.445, giving R squared = 0.20. With this as input the estimated power is 0.75 with 150x2 participants, and the estimated sample size to obtain 80% power is 169x2. But this estimate is also slightly conservative, since we also plan to adjust for the following covariatres: age, dependency level, hospital site, gender, and a measure of stroke severity. This is expected to give a higher R squared than adjusting for baseline mRS alone, and hence give a slightly increased power.

Region:	Saksbehandler:	Telefon:	Vår dato:	Vår referanse:
REK midt	Magnus Alm	73559949	22.01.2019	2018/1809/REK midt
			Deres dato:	Deres referanse:
			08.01.2019	

Vår referanse må oppgis ved alle henvendelser

Torunn Askim
NTNU

2018/1809 Langtidsoppfølging etter hjerneslag. LAST-long studien

Forskningsansvarlig: Norges teknisk-naturvitenskapelige universitet, St. Olavs Hospital HF, Akershus universitetssykehus HF
Prosjektleder: Torunn Askim

Vi viser til søknad om forhåndsgodkjenning av ovennevnte forskningsprosjekt. Prosjektet ble første gang vurdert av komiteen i møtet 31.10.2018. Vedtak ble da utsatt i påvente av en tilbakemelding fra søker. Tilbakemelding ble mottatt 08.01.2019 og er vurdert av komiteens representant for etikk på delegert fullmakt fra komiteen.

Komiteens prosjektsammendrag
Hensikten med studien er å undersøke effekten av en ny behandlingsmodell for langtidsoppfølging etter hjerneslag i kommunen på forebygging av funksjonssvikt. Studien er designet som en klinisk randomisert studie, der intervensjonsgruppen i tillegg til ordinær oppfølging blir fulgt jevnlig av en helsekoordinator, mens kontrollgruppen kun får ordinær oppfølging (TAU). Utfallsmålet er grad av fysisk og kognitiv funksjon etter oppfølging. Data: Allerede registrerte opplysninger hentes fra pasientjournal, som kobles med Dødsårsaksregisteret, Norsk pasientregister, og Reseptregisteret. I tillegg innhentes nye data ved bruk av et testbatteri av kliniske tester, samt et spørreskjema. 490 pasienter som blir innlagt med akutt hjerneslag og skrives ut til hjemmet eller med plan om rehabilitering i eget hjem planlegges rekruttert. Samtykke planlegges innhentet.

Oppsummering av prosjektleders tilbakemelding

Spørreskjemaene
Det var uklart for komiteen om det er forskningsdeltakerne eller lege som skal fylle ut skjemaene. Komiteen mener det vil være for vanskelig for en lekperson å fylle ut skjemaene alene. Prosjektleder opplyser om at alle spørreskjema vil bli fylt ut sammen med studiesykepleier.

Samarbeid med utlandet
Det var uklart for komiteen om studien innebærer samarbeid med forskere i Australia, og land innenfor EU/EØS. Prosjektleder opplyser om at det ikke er snakk om et konkret samarbeid med utenlandske forskere på nåværende tidspunkt, men at man ønsker å innhente samtykke for å kunne benytte data i fremtidige metaanalyser. Prosjektgruppen vil imidlertid søke REK om ny godkjenning før en slik utlevering.

Rekrutteringsprosedyren
Komiteen ba om en presisering av rekrutteringsprosedyren (hvem spør, hvordan, betenkningstid, hvem

svarer de forespurte til). Prosjektleder opplyser om at en invitasjon til studien vil gis til pasientene av behandlende helsepersonell i forbindelse med utskriving fra avdelingen. Et fullstendig informasjonsskriv og forespørsel om samtykke sendes så ut i forbindelse med innkalling til poliklinisk kontroll 3 måneder etter hjerneslaget. Dette vil gi pasientene god tid til å vurdere om de ønsker å takke ja til deltagelse i studien. Sekretær ved poliklinikken vil sikre at alle aktuelle pasienter får tilsendt informasjonsskrivet.

Intervensjonen
Det var uklart for komiteen hva intervensjonen i studien helt konkret innebærer.Prosjektleder gir i tilbakemeldingen en fyllestgjørende beskrivelse av intervensjonen.

Screening
Komiteen ba om en redegjørelse for hvorfor man mener det er nødvendig å screene deltakerne i toomganger. Prosjektleder oppgir at deltagere kun skal screenes en gang, og at det ikke blir foretatt noen screening under sykehusoppholdet.

Vurdering
Komiteen har vurdert tilbakemelding, søknad, forskningsprotokoll, målsetting og plan for gjennomføring. Komiteen finner at prosjektleder har besvart komiteens spørsmål og merknader på en god og fyllestgjørende måte. Komiteen finner at den beskrevne rekrutteringsprosedyren er tilfredsstillende, men noen forutsetninger for godkjenning er spesifisert i avsnittet under. Komiteen vurderer at informasjonsskrivet er endret i henhold til komiteens merknader og har ingen ytterligere innsigelser.

Identifisering av deltakere
Komiteen viser til planlagt prosedyre for identifisering av deltakere. Komiteen forutsetter at det er helsepersonell som allerede er kjent med opplysningene som identifiserer potensielle deltakere. Komiteen viser til hfl. § 35 og innvilger herved dispensasjon fra taushetsplikten slik at helsepersonell kan bruke nødvendig informasjon fra sykehusjournal for å kunne identifisere det aktuelle utvalget og deretter sende informasjonsskriv til vedkommende. De som ønsker å delta, sender så svar til forsker. Forsker får dermed tilgang til personlige helseopplysninger først etter at deltakere har samtykket til dette. Dispensasjonen gis under vilkår om at navnelistene utelukkende benyttes i rekrutteringsøyemed, og at listene slettes når rekrutteringen er gjennomført.

Forsvarlighet
Komiteen har ingen forskningsetiske innvendinger til prosjektet. Under forutsetning av at vilkårene nedenfor tas til følge vurderer REK at prosjektet er forsvarlig, og at hensynet til deltakernes velferd og integritet er ivaretatt.

Vilkår for godkjenning

1. Dispensasjonen fra taushetsplikt gjelder kun for de opplysningene som er relevante for studien.
2. Komiteen forutsetter at ingen personidentifiserbare opplysninger kan framkomme ved publisering eller annen offentliggjøring.
3. Komiteen forutsetter at behandlingen av personopplysninger i forskningen skjer i samsvar med institusjonens retningslinjer for å gi behandlingsgrunnlag. Dette i tråd med personopplysningslovens bestemmelser.
4. Komiteen forutsetter også at prosjektet følger institusjonens bestemmelser for ivaretakelse av informasjonssikkerhet for innsamling, oppbevaring, deling og utlevering av personopplysninger.
5. Av dokumentasjonshensyn skal opplysningene oppbevares i 5 år etter prosjektslutt. Opplysningene skal oppbevares avidentifisert, dvs. atskilt i en nøkkel- og en datafil. Opplysningene skal deretter slettes eller anonymiseres.
6. Prosjektleder skal sende sluttmelding på eget skjema, jf. helseforskningsloven § 12, senest et halvt år etter prosjektslutt.
7. Dersom det skal gjøres endringer i prosjektet i forhold til de opplysninger som er gitt i søknaden, må prosjektleder sende endringsmelding til REK, jf. helseforskningsloven § 11.

Vedtak
Regional komité for medisinsk og helsefaglig forskningsetikk Midt-Norge har gjort en helhetlig forskningsetisk vurdering av alle prosjektets sider og godkjenner det med hjemmel i helseforskningsloven § 10, på de vilkår som er gitt.

Komiteens beslutning var enstemmig.

Merknad
I tilbakemeldingsskjemaet lister prosjektleder opp noen «øvrige endringer». Denne godkjenningen omfatter ikke disse endringene. Komiteen ber prosjektleder søke om disse endringene i en separat prosjektendringssøknad.

Sluttmelding og søknad om prosjektendring
Prosjektleder skal sende sluttmelding til REK midt på eget skjema senest 30.06.2033, jf. hfl. § 12. Prosjektleder skal sende søknad om prosjektendring til REK midt dersom det skal gjøres vesentlige endringer i forhold til de opplysninger som er gitt i søknaden, jf. hfl. § 11.

Klageadgang
Du kan klage på komiteens vedtak, jf. forvaltningsloven § 28 flg. Klagen sendes til REK midt. Klagefristen er tre uker fra du mottar dette brevet. Dersom vedtaket opprettholdes av REK midt, sendes klagen videre til Den nasjonale forskningsetiske komité for medisin og helsefag for endelig vurdering.

Med vennlig hilsen

Vibeke Videm
Professor dr.med. / Overlege
Leder, REK Midt

Magnus Alm
rådgiver

Kopi til:jorunn.helbostad@ntnu.no; Gunnar.Morken@stolav.no; tormod.fladby@medisin.uio.no
