# Supplementary material for: A multimodal individualized long-term intervention to prevent functional decline after stroke (LAST-long): a single blinded randomised controlled trial
Source: Lancet Reg Health Eur. 2025 Nov 13;61:101531. doi: 10.1016/j.lanepe.2025.101531 (PMC12662110; doi:10.1016/j.lanepe.2025.101531)
Supplement: Multimedia component 2 [file mmc2.pdf]

TIDieR

A multimodal individualized long-term intervention to prevent functional decline after stroke (LAST-long)

Why:

Great achievements have been reached within acute stroke treatment over the past decades with strong evidence for treatment in an organized stroke unit and reperfusion therapy for patients with acute ischemic stroke. There is also strong evidence for multidisciplinary person-centred rehabilitation and early supported discharge. In addition, immediate and sustained implementation of effective and appropriate secondary prevention strategies has the potential to reduce the risk of recurrent stroke significantly. Engagement in physical activity is critically important, as structured exercise has dual benefits by both reducing the risk of recurrent stroke and enhancing overall function. Nevertheless, there is substantial variation in adherence to the guidelines, especially in the long term. There is also a need for research on the transition from rehabilitation to life after stroke. Therefore, the Stroke Action Plan for Europe emphasizes long-term follow-up as one of the research priorities toward 2030, and identification of an optimal model of care and long-term support after stroke.

What (material):

The intervention group received 18 monthly meetings with a new established community-based stroke-coordinator in addition to standard care.

A study-specific checklist (the LAST-long checklist) was developed based on the World Stroke Organisation (WSO) Post-Stroke Checklist and Comprehensive Geriatric Assessment (CGA). The original CGA includes four domains of assessment that are; medical assessment, assessment of function, assessment of cognition and mood and social assessment, and is shown to be effective for improving survival and function in older persons. On the other hand, the WSO Post Stroke Checklist, addressing 11 areas, is also aiming to improve long-term stroke care. In the LAST-long checklist, the domains from the WSO Post Stroke Checklist and the CGA were combined to cover all relevant areas for long-term follow-up after stroke.

In the first meeting, the LAST-long checklist was used as a guide for a structured interview to assess the patients' risk profile within the following domains: 1) health and lifestyle, 2) physical function, 3) cognition and mood, and 4) social function. The details of the checklist have been described in the protocol paper and the checklist is attached as supplementary material to this paper (Askim et al., BMJ Open 2023). For patients with risk of deterioration within one or more domains, motivational interviewing and shared decision making was used to set 2-3 achievable individual goals and agree on an appropriate treatment plan aiming to reach those goals. The treatment plan should make use of self management or already available community-based or hospital-based services as recommended by the stroke-coordinator. Within the domain of health and lifestyle factors the goals were based on recommended guidelines, while within the domains of physical function, cognition and mood and social function where no guidelines exists, best evidence and good clinical practice were used to tailor goalsetting and the action plan. In the following meeting, the participant and stroke-coordinator together used a standardised form to evaluate goal achievement and adherence to the treatment plan and a revised plan was set for the next month. This procedure was continued for the next 16 months giving a total of 18 possible meetings per participant.

**What (procedures):**	The stroke-coordinators contacted the participants by phone and scheduled the first meeting within two weeks after randomisation. The first and the last meeting had to be an in-person meeting that could take place in the participants home or at the stroke-coordinator's work place. At least 50% of all 18 meetings should be in-person meetings, while the remaining meetings could be either phone meetings, online meetings or in-person meetings, depending on the participants' preferences. Meetings that occurred during the holiday season might be be cancelled, however the follow-up period was not extended for that reason.
**Who provided:**	The stroke-coordinators who provided the intervention were community based and had a professional background as either physiotherapist, occupational therapist or nurse. The main role of the coordinator was twofold; 1) to set up an individualized treatment plan and 2) to motivate and assist in getting access to the existing services. All coordinators had experience from working in the primary health care system and collaborating closely with the service office, which makes the decision to access the necessary services. Furthermore, the stroke coordinators were certified in motivational interviewing as a technique to facilitate life-style changes and improve adherence to the treatment plan. Regular workshops were arranged for the stroke coordinators to enhance adherence to the intervention protocol.
**How (mode of delivery; individual or group):**	All 18 meetings were individual meetings between the stroke-coordinator and the participants. The first and the last meeting had to be an in-person meeting that could take place in the participants home or at the stroke-coordinator's work place. At least 50% of all 18 meetings should be in-person meetings, while the remaining meetings could be either phone meetings, online meetings or in-person meetings, depending on the participants' preferences. Meetings that occurred during the holiday season might be be cancelled, however the follow-up period was not extended for that reason.
**Where:**	In-person meetings took place in the participants home or at the stroke-coordinator's work place, while other meetings could be either by phone or online.
**When and how much:**	The intervention was delivered as monthly meetings across 18 months. Each meeting lasted for approximately 1 hour.
**Tailoring:**	The intervention was planned to be personalised. The LAST-long checklist was used as a guide for a structured interview to assess the patients’ risk profile. For patients with risk of deterioration within one or more domains, 2-3 individual goals and an individual treatment plan aiming to reach those goals, were set.
**Modification:**	Due to the Covid-19 pandemic we had to make som adjustments by allowing for more meetings to be phone or online meetings than intended for the participants that were recruited during this period.
**How well (planned):**	An attendance rate of 14 out of 18 meetings was defined as good adherence. The adherence was reviewed consecutively by a study coordinator who reviewed the completed checklists, goals and action plans. In addition, regular workshops were arranged to harmonize the intervention across all sites and stroke-coordinators and to ensure good adherence to the protocol. In the workshops we also discussed how to ensure that all domains, also mood, cognition and social function, were assessed equally thoroughly as physical function and physical health.<br><br>Furthermore, goal achievement and adherence to the action plan was evaluated in every meeting unless the first meeting. For this purpose participants were asked to rate on a numeric rating scale (1=fully disagree to 5=fully agree) to which extent they agreed to the following statements; 1) I achieved my goals, 2) I adhered to the action plan.
**How well (actual):**	A total of 96 out of 152 (63 %) participants in the intervention group attended at least 14 meetings. Average goal achievement was 2.9 (SD 1.3), while average adherence to the action plan was 3.7 (SD 0.7).
